# Supplementary material for: Almost all working adults have at least one risk factor for non-communicable diseases: Survey of working adults in Eastern Ethiopia
Source: PLoS One. 2022 Feb 28;17(2):e0264698. doi: 10.1371/journal.pone.0264698 (PMC8884490; doi:10.1371/journal.pone.0264698)
Supplement: Attachment — Submitted filename: Comment.docx [file pone.0264698.s002.docx]

Comment

I am happy to review the manuscript entitled “Almost everyone has at least one risk factor for Non-Communicable Diseases - survey of working adults in Eastern Ethiopi**”** and I would like to thank the esteemed journal to invite me to review the manuscript.

The topic is very important indicating that the magnitude of risk factors of NCDs in the developing world. The aims of the manuscript are clearly defined with concise presentation of results. But I have some concerns that need clarification and which may improve the quality of the document.

1. To attract readers, I would like to suggest you to incorporate the “gap” under introduction of your abstract
2. Is that possible to indicate the available Ethiopian government policies/strategies towards NCDs like that of communicable diseases (under introduction part).
3. Under study setting of your methodology, you mentioned that “The university also runs a specialized referral hospital in Harar town that provides comprehensive health services to the general public” do found that the magnitude of risk factors of NCDs among health professionals is the same to the rest university employees.
4. Under your methods “Data were collected through face-to-face interviews and physical measurement using structured questionnaires adopted from the WHO-STEP survey instrument version 3.1” is the questionnaire validated in the local context or?
5. Under discussion: line 329 & 330“The magnitude of risk factors of NCDs in this study was high among working adults in Ethiopia”, how do you generalize a single institution finding for general Ethiopian working adults?
6. I suggest you to clearly indicate the general implication of your finding, under your discussion as an introduction of the discussion.
7. Figure 1: it is difficult to read the percentages from the figure
8. You have to introduce abbreviations when you use them for the first time (for instance “WHO STEP” under methods of the abstract.
