# Supplementary material for: Almost all working adults have at least one risk factor for non-communicable diseases: Survey of working adults in Eastern Ethiopia
Source: PLoS One. 2022 Feb 28;17(2):e0264698. doi: 10.1371/journal.pone.0264698 (PMC8884490; doi:10.1371/journal.pone.0264698)
Supplement: Attachment — Submitted filename: Response to Reviewers.docx [file pone.0264698.s003.docx]

**To: Almost all working adults have at least one risk factor for Non-Communicable Diseases - survey of working adults in Eastern Ethiopia**

**Subject: Revision and resubmission of manuscript (manuscript ID: PONE-D-21-30898)**

**Dear Editor and reviewers**,

We would like to express our appreciation to the editor and the reviewers for their insightful and constrictive comments on our manuscript entitled “Almost all working adults have at least one risk factor for Non-Communicable Diseases - survey of working adults in Eastern Ethiopia” The comments and suggestions offered by the reviewers have immensely helped us to improve our manuscript.

We have considered each and every comment and have responded point by point, indicating how we addressed them and tracking the changes we have made. The changes are presented in the manuscript. Our responses to the reviewers’ comments are provided below.

We hope the revised manuscript is better suited to your esteemed journal and we are happy to consider any further revision as deemed necessary.

Sincerely,

Aboma Motuma

I. REPLY TO COMMENTS FROM EDITORS

| Comments suggestions | Response |
| --- | --- |
| Please ensure that your manuscript meets PLOS ONE's style requirements, including those for file naming | We checked the guideline and follow all the instruction and standards during the revision |
| We note that you have stated that you will provide repository information for your data at acceptance. Should your manuscript be accepted for publication, we will hold it until you provide the relevant accession numbers or DOIs necessary to access your data. If you wish to make changes to your Data Availability statement, please describe these changes in your cover letter and we will update your Data Availability statement to reflect the information you provide | No change, the data sets analyzed for this manuscript are available from the corresponding author upon reasonable request we will provided the data. |
| One of the noted authors is a group or consortium [Aboma Motuma, Lemma Demissie Regassa, Tesfaye Gobena, Kedir Teji Roba, Yemane Berhane, Alemayehu Worku]. In addition to naming the author group, please list the individual authors and affiliations within this group in the acknowledgments section of your manuscript. Please also indicate clearly a lead author for this group along with a contact email address. | Thank you, there is no group author, but we missed the comma between the names of authors by mistake. This has been changed in the revised manuscript. |
| However, the manuscript has multiple language usage flaws including punctuations, wordings, spelling and mainly grammar errors. These problems are found throughout the manuscript. Therefore, please make repeated proof-reading and thorough copyediting before considering the manuscript for publication. This would help increase the readability of the manuscript if published. | Thank you. Based on the editors and reviewers recommendations, we have gotten a native English speaker to go through the revised manuscript for language clarity. |
| **Specific comments** |  |
| Background of the abstract doesn’t clearly show the existing burden of NCD in in Eastern Ethiopia or other regional states or the country. Generally, burden of NCD should first be stated followed by the objectives showing the research gap the authors would like to address. | Thank you for indicating areas for improvement. We have done significant revisions to the background of abstract and introduction section. The introduction is made to focus on providing the existing burden of NCD in Eastern Ethiopia; what is known and the gaps in knowledge. The aim of the study is also clearly indicated |
| Methods of abstract should include sampling technique, software for data entry and analysis, and cut off P-value to declare statistical significance of factors. | As suggested by the reviewer, a brief description is included in the revised manuscript. |
| Results; kindly include response rate of the study at the beginning. | Based on the recommendations, we have included the response rate in the revised manuscript. |
| Conclusion: The risk score of non-communicable diseases was higher for older and highly educated study participants…The phrase highly educated shall be clearly defined in the methods section. Moreover, kindly make your recommendation specific to your study area than considering the Ethiopian setting. | Thank you for indicating areas for improvement. Based on the recommendations, we revised in the manuscript. |
| Methods  Population and selection criteria  Non-consenting individuals should have been considered as non-respondents than excluding them from the outset. | Thank you, this has been changed in the revised manuscript. |
| How did you identify severe mental disability? | Thank you, this has been changed in the revised manuscript. |
| Sample size and sampling procedure: It would be more self-explanatory and easily understandable if the authors showed pictorial presentation (flow chart) of the sampling procedure including how many campuses, colleges, departments, sample size were considered to reach a response rate of 1,164 (97%). | Thank you for indicating areas for improvement. Based on the recommendations, we revised the manuscript and add figure for sampling procedure. |
| Ethical clearance: What beneficent actions did the authors provide the employees (interviewees) in return for the interviews? | An explanation was given to the study participants about the study procedure, risk, and benefit. Depending on the objective of the study, people with high risk for NCD are benefited from the information dissemination after publication, benefit from annual research conference presentation in the university, discussing on the findings and advised the participants to reduce risk factors to prevent NCDs. Furthermore individuals with high risk factors were linked to the university clinic for further consulting and screening. |
| Discussion and conclusions  Recommendations should be specific and feasible in the given context of the study area. | Thank you, this has been changed in the revised manuscript. |
| **Reviewer #1 Evaluation** |  |
| Comment 1. To attract readers, I would like to suggest you incorporate the “gap” under introduction of your abstract | Thank you for indicating areas for improvement. Based on the reviewer recommendations, we revised the manuscript. |
| Comment 2. Is that possible to indicate the available Ethiopian government policies/strategies towards NCDs like that of communicable diseases (under introduction part). | This is well taken. We accept the comment and have revised, and included the National strategic action plan (NSAP) for prevention and control of Non-communicable disease in Ethiopia, 2014-2016. |
| Comment 3. Under study setting of your methodology, you mentioned that “The university also runs a specialized referral hospital in Harar town that provides comprehensive health services to the general public” do found that the magnitude of risk factors of NCDs among health professionals is the same to the rest university employees. | Thank you. The comment is valid. We included all the working adults including the health workers, there is no significant difference by professional. |
| Comment 4. Under your methods “Data were collected through face-to-face interviews and physical measurement using structured questionnaires adopted from the WHO-STEP survey instrument version 3.1” is the questionnaire validated in the local context or? | Thank you. We adopted the survey as per the national (Ethiopian NCD survey-2015). Since the survey included all adult participants from all enumeration areas in Ethiopia (including our study area) the tools are appropriate to use to assess the NCD risk factors in our setup. In addition, we adopted and modified WHO STEPS survey of NCDs among Bank Employees and Teachers in Addis Ababa, Ethiopia in 2010. |
| Comment 5. Under discussion: line 329 & 330“The magnitude of risk factors of NCDs in this study was high among working adults in Ethiopia”, how do you generalize a single institution finding for general Ethiopian working adults? | Thank you, the comment is valid. This has been changed in the revised manuscript. We considered Haramaya university workers are engaged in a variety of work conditions, from simple labor work to high-level academic works. However, we added statement in the limitation section to indicate wider interpretation shall be made cautiously and thus the findings cannot be fully generalizable to all working adults in Ethiopia. |
| Comment 6. I suggest you to clearly indicate the general implication of your finding, under your discussion as an introduction of the discussion. | Thank you, this has been considered in the revised manuscript. |
| Comment 7. Figure 1: it is difficult to read the percentages from the figure | We accept the comment and figure is corrected as per the standard |
| Comment 8. You have to introduce abbreviations when you use them for the first time (for instance “WHO STEP” under methods of the abstract. | We accept the comment and corrected in the revised manuscript. |
| **Reviewer #2 Evaluation** |  |
| Comment 1. Title : rewrite it as follows: Almost all working adults have at least one risk factor for Non-Communicable Diseases | Thank you. We accept the comment and corrected |
| Comment 2. Abstract  Method  WHO STEP in line 26 should be spell out before abbreviation. | This is well taken. We accept the comment and have revised. |
| Comment 3. Result  Line 33. Show the result with 95% CI | We accept the comment and Corrected |
| Comment 4. Rewrite this statement on line 37 and 38 as follow  “Higher risk factor scores were associated with advanced age (AIRR: 1.24; 95%CI: 1.01, 1.53 in 35-44 age group and AIRR: 1.28; 95%CI: 1.01, 1.62 in 45-54 age group), and high educational level (AIRR: 1.23; 95%CI: 1.07, 1.43 for those completed secondary school and AIRR: 1.29; 95%CI: 1.11, 1.50 for those completed college). | Thank you. We accept the comment and have revised our statement. |
| Comment 5. Conclusion section: rewrite the sentence on line 43 “highly educated study participants” in more plausible words like those who completed high level education. | This is well taken and corrected accordingly |
| Comment 6. Introduction  The statement on line 58 &59 “This 67% increases in partly due to lifestyle change and ageing in sub-Saharan Africa” is not clear; re write it with more clear expression. | This is well taken and corrected accordingly |
| Comment 7. Methods  In line 152-15 what do you mean by co-variate? Are all mentioned co-variate were sued for analysis? If not why you name the as covariate. Age and service of year were indicated in this section; do not you think they can be correlated? | Thank you, this has been changed in the revised manuscript. Before fit into the multivariable analysis in the final model, we have checked the multicolenarity. We couldn’t find a correlation between the two variables (age vs. service of year). |
| Comment 8. Using Ethnicity is not recommended to be used as covariate. | Studies reported as the difference was seen among different ethnicities in different countries (Teh JK, et, al, 2014, Lu HT, et al, 2013). Hence inclusion of ethnicity could does not alter the finding but show its role in prevention of NCDs. However, the ethnicity did not show significant association and one can understand that intervention can be planned irrespective of ethnicities. |
| Comment 9. The statement under “Variables and measurements” is too long. Please only elaborate the measurement you used for your outcome. Avoid every detail in this section. If you are focusing on the current practice you should only elaborate in that context. Most of the statement and elaboration you made here are not useful. | Thank you, this has been changed in the revised manuscript. |
| Comment 10. Data management and analysis  This section lacks model fitness test with its statistic output, and you did not mention the multi-collinear variable. If you did not find any multi collinearity it is good to mention it. | Thank you, these have been included in the revised manuscript. We have checked the multi-collinearity among the independent variables, we didn’t find any multi-collinearity. |
| Comment 11. Results  Line 252. Socio-demographic participant’s characteristics “re write as Socio-demographic characteristics participants” | Thank you, this has been changed in the revised manuscript. |
| Comment 12. Under socio demographic characteristics: avoid writing everything one by one; please focus on the important variable | Thank you for the feedback, we corrected as per your comment. |
| Comment 13. Line 270: Legend EB, should be spell as ETB. | Thank you, this has been changed in the revised manuscript. |
| Comment 14. Line 275: Magnitude of core risk factors. It is highly recommended if you describe your result with 95% CI. | Thank you, this has been changed in the revised manuscript. |
| Comments 15. What is the importance of table 2? Why had you put your outcome variable with different variable? This section need more work. Please clearly show your outcome variable boldly. If you do not have a convincing reason delete table two and show your outcome variable in graph. | Thank you for the suggestion. We omitted table 2 as we are convinced by your feedback that the narration and figure 2 are enough to describe the risks for NCDs. |
| Comment 16. Line 305 Determinants of risk factors? Is determinant factor or associated factor is correct? Please take care of the word you used when you explain the association. I urge you to see this section again. | We assessed the associated factors as the design was cross sectional study. Thank you, this has been changed in the revised manuscript. |
